# Evaluation of the Efficacy of a Cholera Toxin-Based *Staphylococcus aureus* Vaccine against Bovine Intramammary Challenge

**DOI:** 10.3390/vaccines9010006

**Published:** 2020-12-24

**Authors:** Hussain A. Alabdullah, Elise Overgaard, Danielle Scarbrough, Janet E. Williams, Omid Mohammad Mousa, Gary Dunn, Laura Bond, Mark A. McGuire, Juliette K. Tinker

**Affiliations:** 1 Department of Animal and Veterinary Science, University of Idaho, Moscow, ID 83844, USA; hussain.alabdullah@wsu.edu (H.A.A.); janetw@uidaho.edu (J.E.W.); mmcguire@uidaho.edu (M.A.M.); 2 Biomolecular Sciences Graduate Program, Boise State University, Boise, ID 83725, USA; eliseovergaard@u.boisestate.edu (E.O.); danielleholt@u.boisestate.edu (D.S.); 3 Department of Biological Sciences, Boise State University, Boise, ID 83725, USA; omidmohammadmousa@u.boisestate.edu (O.M.M.); gary.dunn@student.montana.edu (G.D.); 4 Biomolecular Research Center, Boise State University, Boise, ID 83725, USA; LBOND@boisestate.edu

**Keywords:** *Staphylococcus aureus*, vaccine, bovine, mastitis

## Abstract

*Staphylococcus aureus* (*S. aureus*) is a primary agent of bovine mastitis and a source of significant economic loss for the dairy industry. We previously reported antigen-specific immune induction in the milk and serum of dairy cows following vaccination with a cholera toxin A_2_ and B subunit (CTA_2_/B) based vaccine containing the iron-regulated surface determinant A (IsdA) and clumping factor A (ClfA) antigens of *S. aureus* (IsdA + ClfA-CTA_2_/B). The goal of the current study was to assess the efficacy of this vaccine to protect against *S. aureus* infection after intramammary challenge. Six mid-lactation heifers were randomized to vaccinated and control groups. On days 1 and 14 animals were inoculated intranasally with vaccine or vehicle control, and on day 20 animals were challenged with *S. aureus*. Clinical outcome, milk quality, bacterial shedding, and somatic cell count (SCC) were followed for ten days post-challenge. Vaccinated animals did not show signs of clinical *S. aureus* mastitis and had lower SCCs compared to control animals during the challenge period. Reductions in bacterial shedding were observed but were not significant between groups. Antibody analysis of milk and serum indicated that, upon challenge, vaccinated animals produced enhanced IsdA- and ClfA-CTA_2_/B specific immunoglobulin G (IgG) responses, while responses to CTA_2_/B alone were not different between groups. Responses after challenge were largely IgG1 against the IsdA antigen and mixed IgG1/IgG2 against the ClfA antigen. In addition, there was a significant increase in interferon gamma (IFN-γ) expression from blood cells in vaccinated animals on day 20. While preliminary, these findings support evidence of the induction of active immunity by IsdA + ClfA-CTA_2_/B, and further assessment of this vaccine is warranted.

## 1. Introduction

Mastitis, or inflammation of the udder, is one of the most economically-significant diseases affecting dairy cattle worldwide and is most often the result of a bacterial infection. *Staphylococcus aureus* (*S. aureus*), a main etiological agent, is highly contagious and can spread rapidly among herds. It is estimated that up to 70% of U.S. herds are positive for *S. aureus,* and this bacterium caused the highest overall annual yield losses among other mastitis pathogens in a recent Finnish study [1,2]. *S. aureus* infections are most commonly transmitted during the milking process and can impact animal welfare as well as milk yield and quality [3]. The ability of this bacterium to form biofilms and replicate intracellularly can promote subclinical colonization of the mammary gland, often leading to chronic infection, which is difficult to detect and is frequently the source of herd re-infection [4,5,6]. *S. aureus* is also commonly resistant to antimicrobial treatment and has a low expected cure rate during lactation [7]. While the impact of *S. aureus* infection is difficult to quantify, clinical mastitis caused by Gram-positive pathogens is reported to cost between $133 and $444 per case, or as much as USD 2 billion annually [8,9]. These costs include many factors such as milk loss, veterinary expenses, diagnostic testing, and loss of animals. Prevention of *S. aureus* mastitis with a cost-effective vaccine would improve animal welfare, reduce antibiotic use, and positively impact the economics and efficiency of milk production.

Previous approaches to *S. aureus* vaccination in cattle include whole-cell live and killed vaccines as well as purified antigens. Currently, two whole-cell inactivated vaccines are licensed for protection against *S. aureus* mastitis—Lysigin^®^ (Boehringer Ingelheim, Duluth, GA, USA) and Startvac^®^ (Hipra, Girona, Spain). While efficacy studies are somewhat conflicting, these vaccines have reported moderate decreases in the incidence of new *S. aureus* intramammary infection but are not in widespread use [10,11,12,13,14,15]. Recent studies have focused on the use of multiple purified surface adhesins and secreted virulence factors to develop a vaccine that offers more strain-to-strain cross-protection. Iron-regulated surface determinant A (IsdA) is a fibrinogen- and fibronectin-binding adhesin that contributes to iron sequestration and is a well-studied *S. aureus* vaccine candidate [16,17,18,19]. The presence of *isdA* is conserved among bovine *S. aureus*, and IsdA is expressed from these strains in milk [18,20,21,22,23]. The clumping factor A (ClfA) fibrinogen adhesin is also highly conserved, expressed from bovine clinical isolates, and a recognized vaccine candidate against mastitis [24,25,26,27,28,29,30]. The conservation, surface exposure, and importance in multiple mechanisms of pathogenesis supports the inclusion of the IsdA and ClfA antigens in a multivalent bovine vaccine. However, a number of additional antigens have been characterized and may be necessary to protect against multiple *S. aureus* serotypes.

While immune correlates of protection are not known, an understanding of immune responses is needed to inform antigen selection. The induction of both humoral and cellular immunity is essential to combating intracellular *S. aureus* infection [31,32,33]. Cellular subpopulations that play a central role in defense against *S. aureus* include neutrophils, CD8^+^ T lymphocytes, and CD4^+^ Th17 lymphocytes [34,35]. Cholera toxin (CT), produced by the bacterium *Vibrio cholerae*, and the homologous heat-labile toxin I (LTI), produced by the bacterium *Escherichia coli*, are gold standard vaccine adjuvants that can stimulate systemic immunity from mucosal and dermal sites (reviewed in [36]). The mechanism of adjuvanticity of these toxins depends upon active binding subunit targeting of dendritic cells and neutrophils, and has been attributed to enhanced antigen presentation, upregulation of surface molecules, and promotion of B-cell isotype switching to antigen-specific immunoglobulin A (IgA) and immunoglobulin G (IgG) [37,38,39,40,41]. CT and its non-toxic binding subunit (CTB) can also induce Th1, Th2, and Th17 responses [42,43,44].

The toxic A subunit of CT (CTA) is subdivided into an enzymatically-active domain (CTA_1_) and a linker domain (CTA_2_), which is non-covalently associated with the B subunit. CTA_2_/B chimeras were first described as a mechanism to make stable human vaccines with antigens coupled to the CTB subunit via the A_2_ linker domain [45,46]. These non-toxic molecules retain the adjuvanticity of CTB and possess additional advantages including ease of purification, direct association of antigen to adjuvant, and a holotoxin-like structure that retains binding and internalization motifs [47,48]. As reported previously, we have incorporated *S. aureus* IsdA and ClfA into a CTA_2_/B vaccine platform (IsdA + ClfA-CTA_2_/B). After two intranasal doses this vaccine was found to stimulate significant *S. aureus* antigen-specific humoral and cellular immunity in bovine blood and milk [49].

For this study we hypothesized that intranasal IsdA + ClfA-CTA_2_/B would be effective in reducing or eliminating *S. aureus* shedding and disease after intramammary challenge in cattle. We describe a preliminary trial to determine the efficacy of this mucosal enterotoxin-based vaccine to protect against acute *S. aureus* mastitis. While the vaccine did not prevent bacterial shedding after challenge, results indicate that IsdA + ClfA-CTA_2_/B induces antigen-specific immune responses that may contribute to a reduction in clinical severity and infiltration of leukocytes, or SCC, in infected animals.

## 2. Materials and Methods

### 2.1. Bacterial Strains, Plasmids, and Growth Conditions

**Figure 1:**
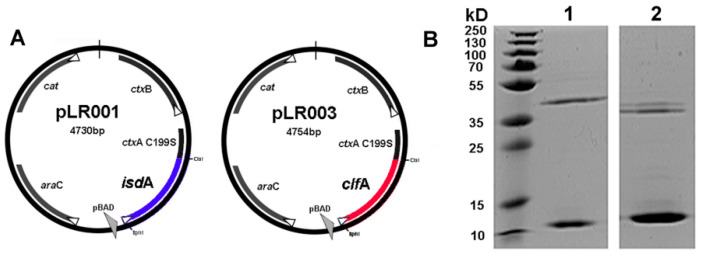
*S. aureus* cholera toxin A_2_/B (CTA_2_/B) chimeric mucosal vaccines. (**A**) pLR001 for expression of IsdA-CTA_2_/B, and pLR003 for expression of ClfA-CTA_2_/B, and (**B**) SDS-PAGE of purified IsdA-CTA_2_/B (1, IsdA-CTA_2_~38 kD, CTB~11 kD) and ClfA-CTA_2_/B (2, ClfA-CTA_2_~37 kD, CTB~11 kD).

*S. aureus* Newbould 305 was used for the cloning of *isdA* and *clfA* to construct IsdA + ClfA-CTA_2_/B and was also used for bacterial challenge [22,50]. *E. coli* ClearColi^®^ (Lucigen, Madison, WI, USA) was used for protein expression (Table 1). The vector pARLDR19 expressing CTA_2_/B and containing a multiple cloning site was used to construct the plasmids pLR001 for Isd-CTA_2_/B expression and pLR003 for ClfA-CTA_2_/B expression (Figure 1A) as described previously [51]. For bacterial challenge, *S. aureus* Newbould 305 was prepared as described [10]. Briefly, Newbould 305 was grown at 37 °C with shaking to mid-log phase in brain–heart infusion and harvested by centrifugation at 3000× *g* for 15 min at 4 °C. The cell pellet was washed with phosphate-buffered saline (1X PBS, pH 7.2) and adjusted to an optical density (O.D.) of 0.2 at 600 nm. Serial dilutions were performed in 1X PBS to reach a bacterial concentration of 400 CFU/mL, as determined by plating on blood agar (BA).

### 2.2. Protein Expression and Purification

Chimeras were purified as previously described [49,51]. Briefly, to express IsdA-CTA_2_/B and ClfA-CTA_2_/B, ClearColi^®^ with pLR001 or pLR003 were grown at 37 °C to an O.D. of 0.9 at 600 nm and induced for 24 h with 0.2% L-arabinose. Proteins were isolated from the periplasmic extract with 1 mg/mL polymyxin B and purified by affinity chromatography on immobilized D-galactose (Pierce™ D-Galactose Agarose, Thermo Fisher, Waltham, MA, USA). Vaccine proteins were dialyzed into sterile 5% glycerol + 1X PBS and concentrations were determined by bicinchoninic acid assay (BCA) (Pierce™ BCA, Thermo Fisher, Waltham, MA, USA). Sizes and purities of the vaccine chimeras were confirmed by sodium dodecyl sulphate-polyacrylamide gel electrophoresis (SDS-PAGE) prior to mixing at a final protein concentration of 600 µg/5 mL for vaccination (Figure 1B). Vaccines were tested to ensure endotoxin levels were below 0.05 EU/mL (LAL Endpoint Chromogenic, Lonza, Allendale, NJ, USA), plated for sterility on tryptic soy agar, and stored at −80 °C until use.

### 2.3. Animals, Vaccination, Challenge, and Clinical Assessment

**Figure 2:**
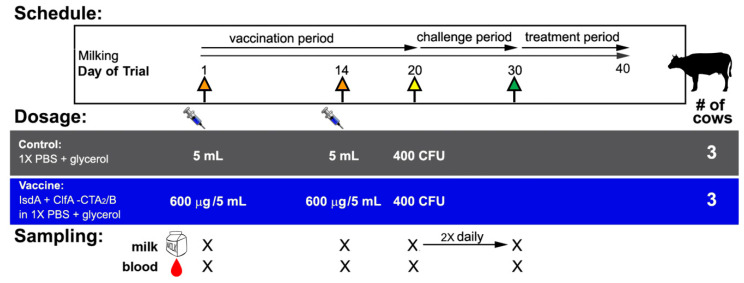
Trial design summary. Animals (*n* = 3 per group, #) were vaccinated intranasally on day 1 and boosted on day 14 with 5 mL of either phosphate-buffered saline (PBS) + 5% glycerol vehicle control or 600 µg IsdA + ClfA-CTA_2_/B vaccine (orange arrows). On day 20 animals were challenged once with 400 colony-forming units (CFU) of *S. aureus* Newbould 305 in two quarters (yellow arrow) and on day 30, animals were treated (end of challenge period, green arrow). Samples of blood were taken on days 1, 14, 20, and 30 (X). Samples of milk were taken on days 1 and 14 (X), and every day for ten days over the challenge period (days 20–30, X→X).

**Figure 3:**
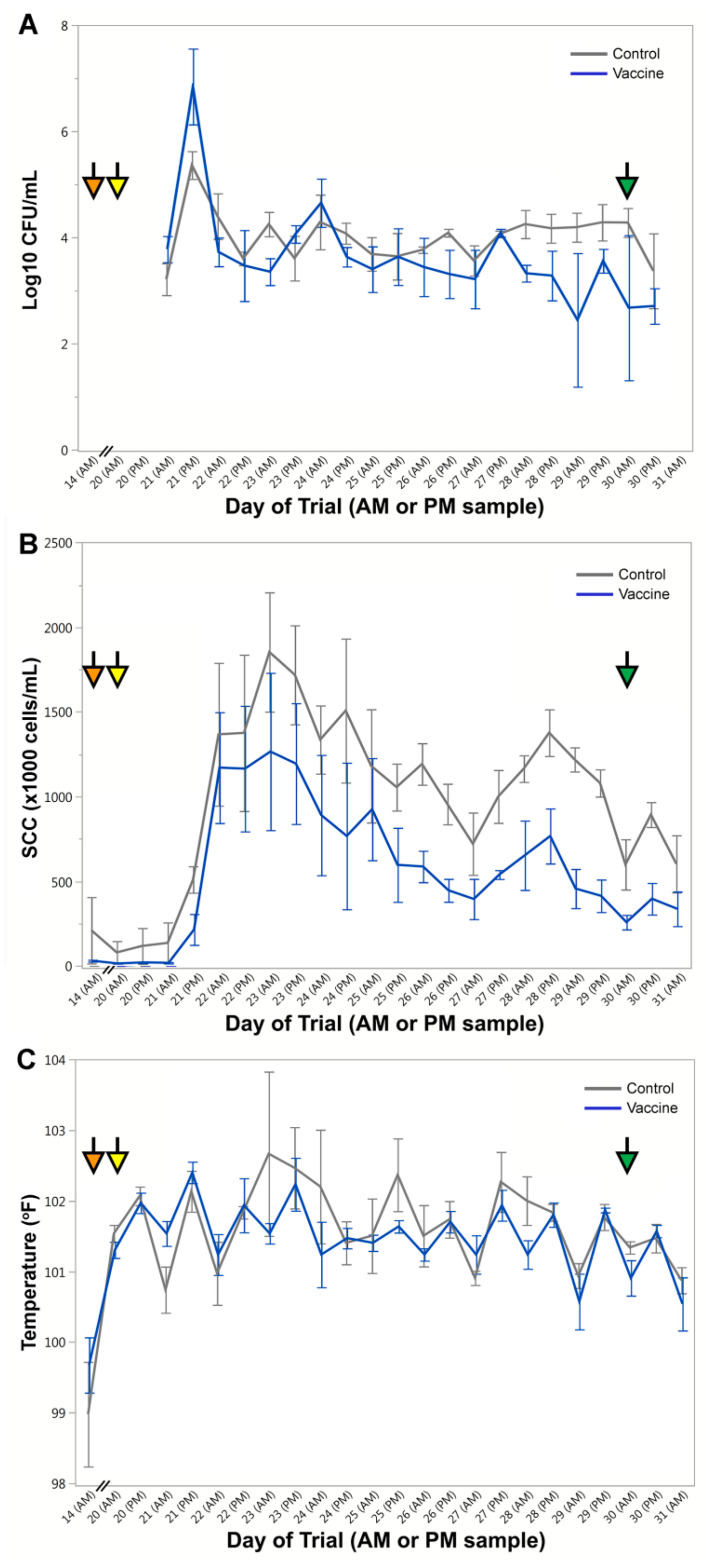
Vaccination outcomes during the trial period. (**A**) Quantification of bacterial shedding by cows during the challenge period. Log10 of CFU/mL of *Staphylococcus aureus* on mannitol salt agar (MSA). Mean ± standard error, *n* = 3 per group, and analyzed using repeated measures analysis of variance (ANOVA). No significance after false discovery rate (FDR) adjustment for multiple comparisons. (**B**) Somatic cell count (SCC) (×1000 cells/mL) by cow. Mean ± standard error, *n* = 3 per group, and analyzed using repeated measures ANOVA. During the challenge period, control cows uniformly had higher SCC than vaccinated cows (main model effect *p* = 0.002). (**C**) Rectal temperature in degrees Fahrenheit (°F). Mean ± standard error, *n* = 3 per group, and analyzed using repeated measures ANOVA showing no significance between groups. Orange arrows indicate day of booster vaccination (14), yellow arrows indicate day of bacterial challenge (20) and green arrows indicates the last day of challenge (30).

All animal protocols were pre-approved by the University of Idaho Animal Care and Use Committee. Lactating healthy Holstein cows in the third or fourth lactation were pre-screened for inclusion as being those with two consecutive SCC readings below 200 × 10^3^ cells/mL and no clinical evidence of mastitis. Further enrollment criteria were followed as described previously [49] and included: (1) no growth of *S. aureus* culture from milk as determined by plating on mannitol salt agar (MSA) and PCR with *S. aureus nuc* and *isdA* primers, (2) low baseline anti-IsdA responses as determined by enzyme-linked immunosorbent assay (ELISA) of milk and serum, and (3) no evidence of bovine leukemia virus infection (Washington Animal Disease Diagnostic Lab, WADDL, Pullman, WA, USA). Seven selected cows were ultimately randomized into vaccinated and control groups. Figure 2 shows the summary of trial design. Four vaccinated animals received a 600 µg intranasal dose of IsdA + ClfA-CTA_2_/B in 1X PBS + 5% glycerol on days 1 and 14 (orange arrows, blue bar), and a control group of three animals of similar age and lactation period received vehicle control (1X PBS + 5% glycerol) mock vaccination on days 1 and 14 (orange arrows, grey bar). The vaccine was delivered in 2.5 mL volumes into each nare using a nasal cannula (Merck & Co., Kenilworth, NJ, USA). On day 20, all animals were challenged in two quarters with 400 CFU in 1 mL of *S. aureus* Newbould 305 (yellow arrow). Quarters were identified as left front (LF), left rear (LR), right front (RF), and right rear (RR). The bacterial challenge was inoculated into two diagonal quarters of each vaccinated cow using teat cannulae (Valley Vet Supply, Marysville, KS, USA). Animals were monitored closely during the challenge period (days 20 to 30) and evaluated for the presence of clinical mastitis by assessment of rectal temperature (Figure 3), milk quality (Figure S2), and udder consistency (examination for edema, hardening, and/or swelling, Table S1) on days of milk sampling during the trial (Figure 2) [54,55]. Enrolled animals that developed pain and/or fever that exceeded 103 °F were administered painkillers (Banamine^®^ and aspirin) as recommended by the attending veterinarian. Shortly after challenge (day 21), one vaccinated animal developed a severe *Escherichia coli* mastitis case in an unchallenged quarter (2779 LF) with systemic illness including septicemia. Thereafter, the other three quarters were involved and the animal developed clinical mastitis due to *Staphylococcus aureus*. The animal was euthanized on day 5 post-challenge. Results from this animal are not included in the data in this report and resulting sample size was *n* = 3 per group, as represented in Figure 2. On day 30 all other animals began treatment with Spectramast (Zoetis, Parsippany, NJ, USA) until consecutive negative cultures were indicative of safe release to herd as determined by the attending veterinarian.

### 2.4. Sample Collection and Milk Culture

Blood and milk were sampled on day −2 for screening and then on days 1, 14, 20, and 30, and milk was sampled twice daily during the challenge period (Figure 2). Blood was collected from the tail vein and allowed to coagulate at room temperature (RT) for 1 h prior to centrifugation and resuspension into 1:10 inhibitor solution (IS, 1X HALT™ protease inhibitor and 5% glycerol in 1X PBS). On day 20, whole blood was also collected in vacutainer tubes for peripheral blood mononuclear cell (PBMC) isolation (Becton Dickinson, Franklin Lakes, NJ, USA). Milk was collected aseptically as 50 mL quarter samples after washing teat ends with 70% ethanol and was aliquoted into three equal tubes for culture, SCC, and ELISA. For SCC, milk was fixed prior to shipping and analysis was performed using the California Mastitis Test (WADDL, Pullman, WA, USA). For ELISA, milk was centrifuged at 700× *g* for 20 min at 4 °C to remove fat. Skim milk was collected and centrifuged at 20,000× *g* for 30 min at 4 °C. Whey was collected and stored in 1:10 IS. Equal volumes of diluted whey from each quarter were pooled and stored at −20 °C prior to analysis. For milk culture, 100 µL and 10 µL of tenfold serially-diluted quarter milk was plated on MSA, BA, and MP2 agar (Udder Health Systems, Inc., Meridian, ID, USA) to determine the number of colony-forming units per mL (CFU/mL). The presence of larger yellow colonies with yellow zones on MSA, beta-hemolysis on BA, or small, white, esculin-negative colonies on MP2 was considered presumptive *S. aureus*. These colonies were isolated and confirmed by a positive coagulase test or a PCR test using *nuc* or *isdA* primers [23]. CFU by quarter data, based upon final quantitation on MSA, was determined once daily on days −2, 1, 14, and 20 prior to challenge and twice daily (AM/PM) during the challenge period. Quarter data were combined and total CFU/mL by cow was reported for six animals (*n* = 3 per group).

### 2.5. IgG, IgG1, IgG2, and IgA Enzyme-Linked Immunosorbent Assay (ELISA)

IsdA- and ClfA-specific immune responses in serum and milk were detected using ELISA as described [51]. Briefly, 96-well microtiter plates (Nunc, Thermo Fisher, Waltham, MA, USA) were coated with 10 µg of either IsdA-CTA_2_/B, ClfA-CTA_2_/B, or CTA_2_/B in 1X PBS and incubated overnight at 4 °C. Coated plates were blocked for 2 h at 37 °C in 1% goat milk + 1X PBS. After washing, plates were incubated with two-fold dilutions of either bovine serum (dilutions initiated at 1:200 concentration) or pooled quarter milk (dilutions initiated at a 1:10 concentration). Plates were incubated at 4 °C overnight. After washing, plates were incubated with horseradish peroxidase (HRP)-conjugated anti-bovine IgG, IgG1, IgG2, or IgA (1:10,000 Bethyl Laboratories, Montgomery, TX, USA) at 37 °C for 1 h. Plates were developed with tetramethylbenzidine (Promega^TM^ TMB One, Thermo Fisher, Waltham, MA, USA) and read at 370 nm per TMB manufacturer’s instruction. ELISA results from serum or pooled quarter milk were reported by cow (*n* = 3) and presented as the ratio of results (day X/day 1) of the O.D. (370 nm) from a representative antibody dilution in the linear part of the curve (1:1600 serum, 1:160 milk). Results are the average of three independent assays.

### 2.6. Peripheral Blood Mononuclear Cell (PBMC) Isolation and Cytokine qPCR

PBMCs were isolated from whole bovine blood on day 20 for cytokine analysis. PBMCs were isolated using a density gradient established by layering whole blood diluted 1:2 with 1X PBS on Histopaque^®^-1077 (Sigma-Aldrich, St. Louis, MO, USA). Blood samples were centrifuged at 800× *g* for 30 min at RT. The buffy coat was removed and washed three times by centrifugation with Hank’s Balanced Salt Solution for 10 min at 400× *g* at RT, and cells were counted with 0.2% trypan blue. For cytokine assays, total RNA from PBMCs from each cow (*n* = 3 per group) was extracted (RNeasy, Qiagen, Germantown, MD, USA) with an additional Dnase I (Promega, Madison, WI, USA) digestion. cDNA was reverse transcribed per manufacturer’s instructions (High-Capacity RNA-to-cDNA™ Kit, Thermo Fisher, Waltham, MA, USA). qRT-PCR was conducted using SYBR fast (Kapa Biosystems, Thermo Fisher, Waltham, MA, USA) on interferon gamma (IFN-γ), interleukin-6 (IL-6), and interleukin-10 (IL-10) primers, using bovine glyceraldehyde 3-phosphate dehydrogenase (GAPDH) as a reference gene (primers, Table 1). Results are presented as relative gene expression 2^−ΔΔCt^ [56]. All qRT-PCR experiments were performed in triplicate per cow PBMC sample.

### 2.7. Sample Size, Statistical Methods, and Analysis

Sample size was estimated prior to study by power analysis based upon predicted SCC and CFU/mL in milk and using the assumption that quarters are independent, as has been reported [57,58]. A sample size of 13 quarters per group was predicted to provide, at a 95% level of confidence, 80% power to detect a difference in logged SCC. Resulting quarter bacterial counts and SCC data from this study were analyzed by (1) assuming independent quarters and (2) as the combined average of quarters by cow. Outcomes were not different, thus results are reported as the average by cow and assuming quarters are not independent. The log-base 10 values of CFU, SCC, temperature, and serum and milk anti-IsdA, ClfA, and CTB antibodies were analyzed using repeated measures analysis of variance (ANOVA) with time as the within-subjects variable and group as the between-subjects variable. Within-subjects correlation was modeled with either first-order autoregressive or compound symmetric structure, depending on Akaike’s Information Criterion [59]. Comparisons of interest were identified prior to modeling and were examined regardless of the significance of main effects or interaction. First, we explicitly compared the outcome at each study time point. Second, we examined the change in outcome within group, comparing days and adjusting the paired comparisons using false discovery rate [60]. Cytokine analysis was performed using a two-group t-test between vaccinated and control animals. Statistical analyses were conducted using JMP and SAS software (Cary, NC). *p*-values are reported as *p* ≤ 0.05(*), *p* ≤ 0.01(**), or *p* ≤ 0.0001(****) and reflect two-sided tests.

## 3. Results

### 3.1. Bacterial Culture and Clinical Assessment

Quantification of *S. aureus* was determined after plating milk that had been sampled once daily on days −2, 1, 14, and 20 prior to challenge and twice daily during the challenge period (Figure 2). Prior to challenge no animals were found to be shedding *S. aureus*, and immediately after challenge all animals shed high levels of *S. aureus* from infected quarters (Figure 3A). Results revealed a rapid decline in bacterial shedding from all animals within 24 h and then a slow decline beginning in the middle of the challenge period. Between days 2 and 10 of the challenge period (days 22 and 30 of trial) control animals shed a total of 1.08 × 10^6^ CFU/mL and vaccinated animals shed 7.53 × 10^5^ CFU/mL. Differences between treatment groups were observed on days 21, 29, and 30 during the challenge period (*p* = 0.029, 0.011, and 0.018, respectively), however, after adjusting for multiple comparisons, these results are not significant. *S. aureus* was isolated from all challenged quarters in both treatment groups, and all animals continued to shed *S. aureus* throughout the trial. Animals did not shed from uninfected quarters. While one vaccinated animal was culture negative at two time points late in the challenge period (day 29 AM and day 30 AM), no animals were consistently sterile of *S. aureus* by the end of the challenge period. Analysis of positive quarters indicated that there were more days showing a lower percentage of infected quarters for vaccinated animals (Supplementary Figure S1).

SCC taken once daily before the challenge period and twice daily during challenge is shown in Figure 3B. Results show a consistently reduced SCC from vaccinated animals beginning 48 h post-challenge (day 22). While individual days were not significant after adjustment, across and after the challenge period (days 21 to 39) unvaccinated animals had significantly-higher SCC than vaccinated animals (model main effect of treatment group, *p* = 0.002). SCCs of individual cows throughout the trial are shown in Supplementary Figure S3B.

The average rectal temperature per group during the challenge period is shown in Figure 3C. Temperatures at 72 h post-challenge (day 23 AM) showed an average of 102.7 °F for control and 101.5 °F for vaccinated animals, however there was no statistically-significant difference in temperature between groups on any day during this period. In addition, no differences in temperature between groups occurred within 24 h after vaccination (days 1 and 14).

Clinical assessments indicated that animals did not show signs of systemic illness, loss of appetite, or adverse local reactions due to the vaccine, and no animals had clinical evidence of mastitis prior to challenge (day 20). Clinical results are summarized in Supplementary Table S1. Clinical mastitis due to *S. aureus* was observed in challenged quarters of control cows 2767 (LF and RF) and 2830 (LR) throughout the evaluation period. The latter cow developed a persistent mastitis starting on day 23 with apparent milk changes that included clots and flakes in the LR quarter. Clinical mastitis in this animal included persistent udder swelling in addition to pain, heat, and sensation of the affected teat until the end of the challenge period. Temporary enlargement of the supramammary lymph node was noted in one of the vaccinated cows (2823) on day 24, and persistent enlargement observed in one control animal (2830).

Milk quality assessments indicated that while the fat, protein, lactose, and solids-not-fat (SNF) percentages were frequently higher in vaccinated animals, these differences were not statistically significant (Supplementary Figure S2).

### 3.2. Vaccine-Specific Antibody Responses in Blood and Milk

**Figure 4:**
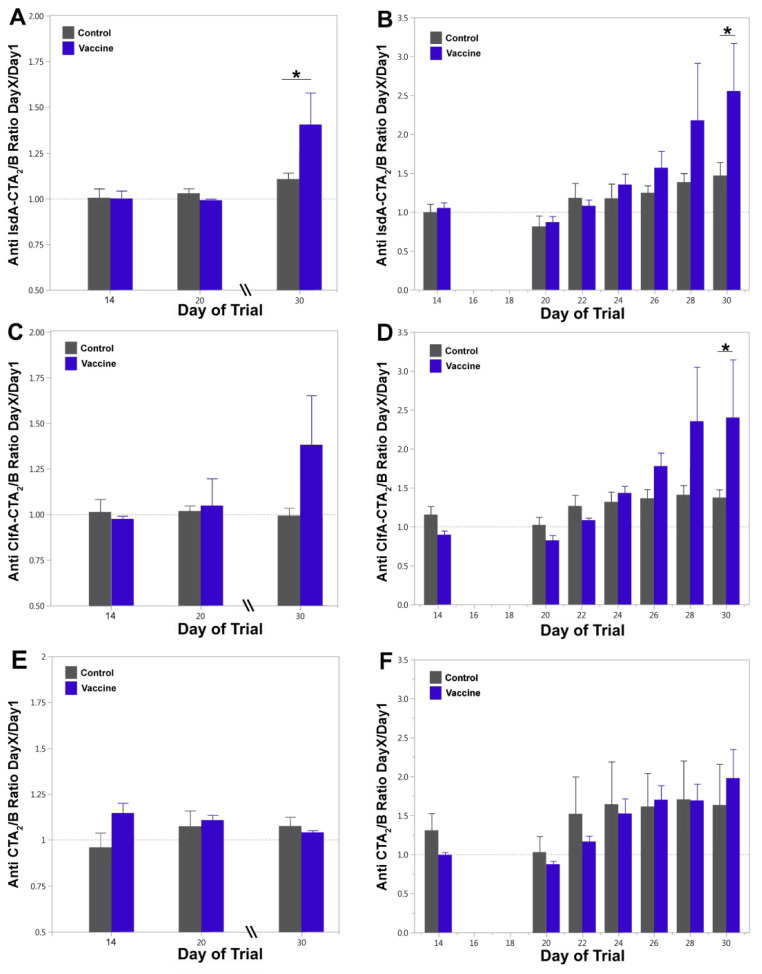
Immunoglobulin G (IgG) antibody responses in serum and milk as determined by enzyme-linked immunosorbent assay (ELISA). Anti-IsdA-CTA_2_/B IgG responses in (**A**) serum and (**B**) milk, anti-ClfA-CTA_2_/B IgG responses in (**C**) serum and (**D**) milk, and anti-CTA_2_/B IgG responses in (**E**) serum and (**F**) milk. Serum was analyzed on days 14, 20, and 30 and milk on days 14, 20, 22, 24, 26, 28, and 30 during the trial period. Results are reported as ELISA ratios of day X/day 1 at O.D. 370 at serum dilutions of 1:1600 and milk dilutions of 1:160. Shown are mean and standard error by treatment with control (gray) and vaccinated (blue) (*n* = 3 per group). Significant differences between groups are represented as *p* ≤ 0.05 (*). The log10 of the values were analyzed using repeated measures analysis of variance (ANOVA) with a compound symmetric covariance structure for cows across days. Model-based estimates were compared between groups within days and adjusted for multiple comparisons.

Antigen-specific humoral responses were quantified by ELISA from blood and milk. Anti-IgG responses in serum on days 14, 20 and 30, relative to day 1, are shown in Figure 4A–C and E. Vaccinated animals (blue bars) showed a significant IsdA-CTA_2_/B-specific IgG response in serum after challenge on day 30 relative to days 14 and 20 (*p*_adj_ = 0.008 for both) and on day 30 relative to control animals (*p* = 0.030 *) (Figure 4A). Vaccinated animals showed a similar, but non-significant, anti-ClfA-CTA_2_/B-specific IgG responses in serum on day 30 relative to days 14 and 20 (*p*_adj_ = 0.120) as well as on day 30 relative to control animals (*p* = 0.079) (Figure 4C). Anti-CTA_2_/B-specific IgG responses in serum remained low and non-significant between groups throughout and after challenge (day 30) (Figure 4E).

Anti-IgG responses in milk on days 14, 22, 24, 26, 28, and 30, relative to day 1, are shown in Figure 4B,D–F. IsdA-CTA_2_/B-specific IgG responses in milk increased over the challenge period in vaccinated animals, with values significantly higher on day 30 relative to days 20 to 26 (adjusted *p*-values all <0.05) and on day 30 relative to control cows (*p* = 0.030 *) (Figure 4B). The anti-IsdA-CTA_2_/B differences between days for control cows were non-significant after day 20. Anti-ClfA-CTA_2_/B-specific IgG responses in milk were significant on day 30 relative to days 20–24 (adjusted *p*-values all <0.05) for the vaccinated group and on day 30 relative to unvaccinated cows (*p* = 0.043 *) (Figure 4D). Milk anti-CTA_2_/B-specific IgG responses increased moderately during the challenge period in both vaccinated and control animals with significant increases on day 30 relative to days 14 and 20 in the vaccinated group and no change in the control group (adjusted *p*-values all <0.05). The differences in anti-CTA_2_B responses between vaccine and control groups were non-significant on all days tested (Figure 4F).

**Figure 5:**
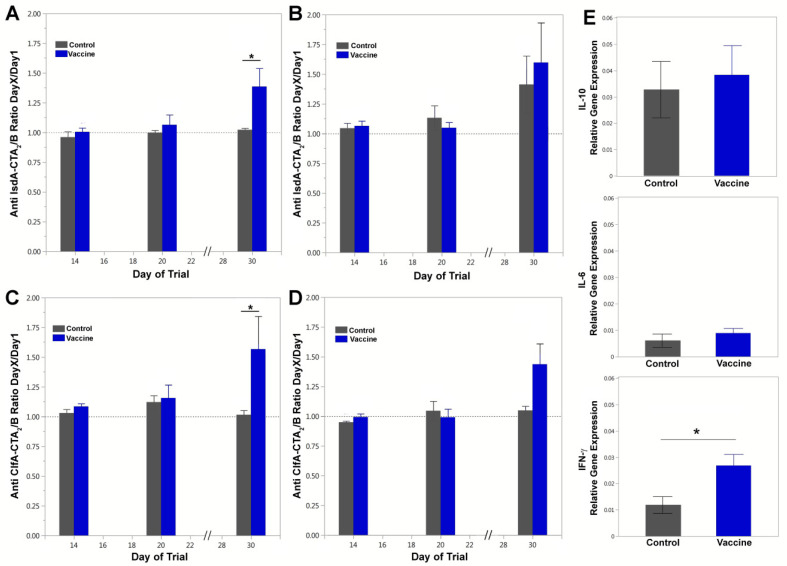
Serum IgG1, IgG2, and cytokine expression analysis. (**A**) Anti-IsdA-CTA_2_/B IgG1, (**B**) anti-IsdA-CTA_2_/B IgG2, (**C**) anti-ClfA-CTA_2_/B IgG1, and (**D**) anti-ClfA-CTA_2_/B IgG2 responses in serum on days 14, 20, and 30. Results are reported as ELISA ratios of day X/day 1 at O.D. 370 at serum dilutions of 1:1600. Shown are mean and standard error by treatment with control (gray) and vaccinated (blue) (*n* = 3 per group). The log10 of the values were analyzed using analysis of variance (ANOVA), with a compound symmetric covariance structure for cows across days. Model-based estimates were compared between groups within days and adjusted for multiple comparisons. (**E**) IL-10, IL-6, and IFN-γ expression as determined by quantitative RT-PCR of peripheral blood mononuclear cells (PBMCs) isolated from whole blood after boost on day 20. Results are shown as relative gene expression to GAPDH (2^−ΔΔCt^). Data are presented as mean and standard error of control (gray) and vaccinated (blue) showing median and range (*n* = 3 per group). Data were analyzed using a two-group *t*-test between vaccinated and control. Significant differences between groups are represented as *p* ≤ 0.05 (*).

Serum IgG subtype (IgG1 and IgG2) responses were evaluated to further define the T helper immune response (Figure 5A–D). Vaccinated animals exhibited increases in IgG1 and IgG2 responses on day 30 for both the IsdA- and ClfA-CTA_2_/B antigens. The serum anti-IsdA-CTA_2_/B IgG1 response on day 30 relative to days 14 and 20 was significant for vaccinated animals (blue bars, *p*_adj_ = 0.004 and *p*_adj_ = 0.007, respectively), and the difference between groups was significant on day 30 (*p* = 0.033 *) (Figure 5A). For anti-IsdA-CTA_2_/B IgG2 responses, day 30 was higher than days 14 and 20 for both vaccinated and control groups (*p*_adj_ = 0.045 for both comparisons) with no significant differences between groups on day 30 (Figure 5B). For serum anti-ClfA-CTA_2_/B IgG1, vaccinated animals showed an increase on day 30 compared to day 14 (*p*_adj_ = 0.023) and day 20 (*p*_adj_ = 0.029), and the difference between groups was significant on day 30 (*p* = 0.029 *) (Figure 5C). For anti-ClfA-CTA_2_/B IgG2 responses, day 30 was higher than days 14 and 20 for both groups as well (*p*_adj_ = 0.015 for both comparisons), however the difference between vaccinated and control groups on day 30 was non-significant after adjustment (*p* = 0.050) (Figure 5D). Assessment of milk anti-IsdA-CTA_2_/B and anti-ClfA-CTA_2_/B IgG1, IgG2 and IgA responses was also performed, and while results indicated an increase on day 30 for both IgG1 and IgA, they were non-significant between vaccine and control groups on the days (14, 20, and 30) tested (data not shown). 

Combined, ELISA analysis shows an induction of antigen-specific humoral responses in the milk and serum after intranasal IsdA + ClfA-CTA_2_/B vaccination, as evidenced by a significant booster effect upon bacterial challenge. Antibody subtyping indicated that both antigens stimulated a Th2-type response, with ClfA potentially inducing a mixed Th1/Th2 response. Lastly, there was no significant antibody response to the CTA_2_/B adjuvant vector alone.

### 3.3. Cytokine Assay

The stimulation of cellular cytokine responses was assessed by quantitative RT-PCR using PBMCs isolated from vaccinated and control cows on day 20 (Figure 5E). IL-12, TNF-α, and IL-4 levels were not significantly different between vaccinated and control animals (data not shown). Vaccinated cows showed a slight but significant increase in IFN-γ expression (*p* = 0.048 *) but no significant difference in IL-10 or IL-6 expression (Figure 5E).

## 4. Discussion

This report describes the outcomes of a small bovine challenge trial to assess the efficacy of the IsdA + ClfA-CTA_2_/B mucosal *S. aureus* mastitis vaccine. We hypothesized that vaccination would prevent or reduce bacterial shedding from the udder after intramammary challenge and reduce disease outcomes. Animals were vaccinated intranasally during milking and challenged in two quarters with the homologous *S. aureus* Newbould 305 vaccine strain. An averaged reduction in CFU/mL from combined quarters of vaccinated compared to unvaccinated animals was observed beginning 24 h after challenge to the end of the challenge period, however, this difference was not significant on specific days during the challenge period. Analysis of bacteriology using independent quarters did not change data interpretations, however, a lower percentage of infected quarters was observed on multiple days after challenge. Analysis of SCC revealed that vaccinated animals had lower numbers of cells on the majority of days during the challenge period of the trial, and this decrease was significant between vaccinated and control animals during the whole of the period. These results were also consistent with the evidence of reduced clinical mastitis in vaccinated animals.

The assessment of humoral immune responses in milk and serum in this report showed induction of IsdA- and ClfA-CTA_2_/B specific IgG antibodies in vaccinated animals after *S. aureus* challenge indicating that vaccination induced antigen-specific responses that were amplified by bacterial challenge. In contrast to previous studies, no significant increase in antigen-specific humoral responses was detected in the serum directly after vaccination and boost, despite the same vaccine dose and schedule [49].The lower sample size in this trial compared to previous trials with IsdA + ClfA-CTA_2_/B may have contributed to this outcome, and larger trials will be essential to advance this vaccine candidate. In addition, animals were vaccinated during milking for this study instead of during dry-off, which is a period of higher susceptibility to mastitis and changes in immune function that may explain observed differences in immunogenicity. As with previous trials, antibody analysis revealed that not all vaccinated animals responded well to the same vaccine preparation and dosage. Variations in host genetics or inconsistencies in administration can cause these disparities, and larger trials will help to exclude them. Other vaccination routes, or alternate prime-boost strategies, may also promote vaccination consistency and efficacy. These routes were not explored in this early study to enable a narrow focus on mucosal delivery, but intramuscular, subcutaneous, and transdermal routes are all effective for CT-adjuvanted vaccines and could be explored. Lastly, in this study we maintained a short dosage interval of only 14 days to align with previous trials, however, a longer interval between doses may improve responses and will be explored in the future.

Animals were vaccinated during milking to permit bacterial quantification and limit the potential for systemic or chronic infection. Despite this, one vaccinated animal was euthanized shortly after challenge due to an *E. coli* infection that rapidly became systemic. While little has been reported about the effects of co-infection on the severity of *E. coli* mastitis, the cow immune status is a key factor, and *S. aureus* is known for the production of virulence factors that modulate the immune response [61]. Specifically the *S. aureus* superantigens (SAgs) can activate specific T-cell subsets, resulting in inflammation, tissue damage, and potential T-cell anergy [62,63,64]. *S. aureus* Newbould 305 strain was chosen for these studies because it induces mild and chronic mastitis, has been utilized before in vaccine challenge trials, and contains a limited repertoire of SAgs [50,65,66]. It is recognized, however, that immune dysregulation likely occurred upon challenge and, despite vaccination, contributed to the enhanced spread and systemic infection in this animal. The potential for co-infection and the ability of the vaccine to protect against heterologous *S. aureus* isolates that may induce more severe disease will both need to be addressed in future studies.

As described above, CT and its non-toxic B subunit can induce humoral and cellular immune responses to co-delivered antigens. CTA_2_/B molecules retain much of the well-characterized adjuvanticity of CTB to induce both humoral and cellular responses. The IgG1 and IgG2 profiles we observed in the serum of vaccinated animals on day 30 were consistent with our previous studies indicating that CTA_2_/B chimeras promote a largely Th2-type cellular response [49,51]. In the current study, however, the responses to IsdA were more clearly polarized toward Th2, while the anti-ClfA responses are supportive of a potential mixed Th1/Th2 response. Cytokine expression analysis in the current study, performed on day 20 prior to challenge, showed no effect on IL-10 and IL-6, but an increase in IFN-γ in vaccinated animals. Cytokine analysis from previous immunogenicity studies using the IsdA + ClfA-CTA_2_/B vaccine largely supported a Th2-type response and did not indicate IFN-γ upregulation [49,51]. This apparent contradiction may be due to differences in the timing of analysis (6 days after vaccination in the current study versus 45 days after vaccination in previous studies) and the methods used (unstimulated versus stimulated PBMCs). Reports indicate that while CTB more commonly induces Th2-type responses, it can induce a mixed Th2/Th1 response with enhanced IFN-γ secretion, depending upon the antigen and route of delivery [42,67,68,69,70,71]. Similar to CTB, vaccination with CTA_2_/B chimeras may promote early macrophage or dendritic cell activation and antigen presentation through IFN-γ upregulation. In this study there was not a clear early effect on the inflammatory and pro-inflammatory balance of serum IL-6 and IL-10, however, others have reported anti-inflammatory properties in CT and its derivatives. These properties may be advantageous for the prevention of *S. aureus* udder colonization and are consistent with our observed reduction in SCC after challenge [72,73,74].

Lastly, in this study we determined if animals responded to the vaccine adjuvant alone by producing anti-CTA_2_/B humoral responses. Results showed no significant differences between vaccinated and control groups. While *S. aureus* challenge was not expected to induce anti-CT antibodies, previous studies have reported the undesirable effect of significant anti-CT antibodies after use of this adjuvant for mucosal vaccination [75]. The low adjuvant-specific antibody response observed here, combined with the reduced recruitment of somatic cells, provides support for the utility of CTA_2_/B-based vaccines.

These studies indicate that IsdA + ClfA-CTA_2_/B may be effective in the reduction of *S. aureus* colonization and clinical outcome, as evidenced by reduced SCC, but do not provide evidence of complete protection or elimination. Both vaccinated and unvaccinated animals shed high levels of *S. aureus* Newbould 305 immediately after challenge, and all animals in the study were found to shed the challenge strain during the entire 10-day challenge period. This outcome may be the result of a high bacterial dose and the artificial nature of intramammary challenge. The use of a lower challenge dose, a different method of challenge, and/or focus on natural transmission in a larger field trial will better determine efficacy to prevent infection. In addition, studies are needed that utilize heterologous isolates, compare outcomes with current vaccines, and assess alternate routes of immunization. IsdA and ClfA are established and highly-conserved antigens from bovine *S. aureus*, however, the incorporation of additional antigens, including toxins and anti-immune factors, may also be necessary to promote strain cross-protection and control immune modulation.

## 5. Conclusions

Results indicate vaccine efficacy in reducing SCC and improving clinical outcome and support further exploration of the IsdA + ClfA-CTA_2_/B vaccine to prevent bovine mastitis. The development of an effective vaccine to prevent mastitis caused by *S. aureus* would have many positive impacts on animal health and food production and may decrease overall antibiotic use in the industry. Needle-free vaccination of cattle would also be beneficial by reducing the transmission of disease, inducing mucosal immunity, and promoting vaccine distribution and use. This study provides important preliminary results of a cholera-toxin-based intranasal vaccine in a mastitis challenge model and supports the continued exploration of this antigen-adjuvant platform to prevent bovine disease.

## Figures and Tables

**Table 1:** Bacterial strains, plasmids, and primers used in this study.

**Bacterial Strains**	**Genotype or Characteristics**		**Source**
*E. coli* ClearColi^®^	BL21(DE3)		Lucigen, Madison, WI
*S. aureus* Newbould 305	Bovine clinical isolate		[50]
**Plasmids**	**Gene**	**Vector**	**Source**
pLR001	*isdA* (Newbould)	pARLDR19	[49]
pLR003	*clfA* (Newbould)	pARLDR19	[49]
**Bovine Cytokine qPCR Primers**	**Gene**	**Amplicon**	**Source**
FW 5′-GCATCGTGGAGGGACTTATGA-3′	GAPDH	67	[52]
RV 5′-GGGCCATCCACAGTCTTCTG-3′			
FW 5′-CTTGTCGGAAATGATCCAGTTTT-3′	IL-10	66	[53]
RV 5′-TCAGGCCCGTGGTTCTCA-3′			
FW 5′-CAGAAAGCGGAAGAGAAGTCAGA-3′	IFN-γ	72	[52]
RV 5′-TGCAGGCAGGAGGACCAT-3′			
FW 5′-GGCTCCCATGATTGTGGTAGTT-3′	IL-6	64	[53]
RV 5′-GCCCAGTGGACAGGTTTCTG-3′			

## Supplementary Materials

The following are available online at https://www.mdpi.com/2076-393X/9/1/6/s1, Figure S1: Percent *S. aureus* Newbould 305 infected quarters from vaccinated and control groups during the challenge period, Figure S2: Milk quality assessment during the trial period, Figure S3: Individual cow bacterial shedding and SCC during trial, Table S1: Clinical outcomes after challenge on day 20.
