# Supplementary material for: Evaluation of the Efficacy of a Cholera Toxin-Based Staphylococcus aureus Vaccine against Bovine Intramammary Challenge
Source: Vaccines (Basel). 2020 Dec 24;9(1):6. doi: 10.3390/vaccines9010006 (PMC7824273; doi:10.3390/vaccines9010006)
Supplement: Supplementary file 1 [file vaccines-09-00006-s001.pdf]

vaccines

Supplementary Materials:

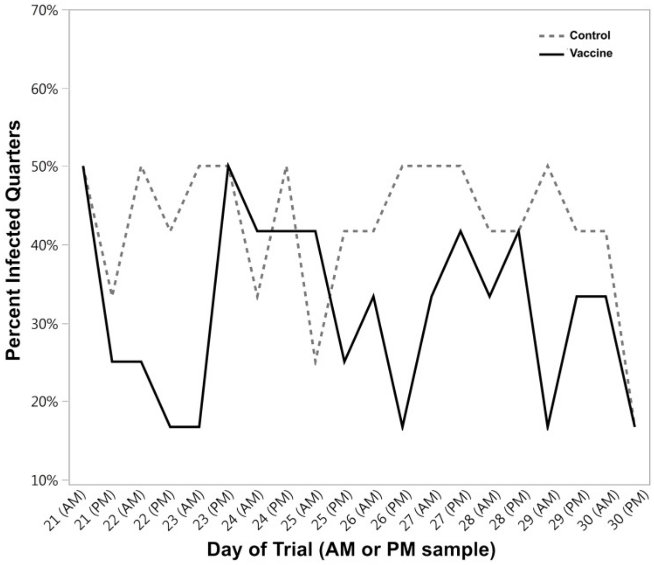

Figure 1. Percent *S. aureus* Newbould 305 infected quarters from vaccinated and control groups during the challenge period (n=12 per group).

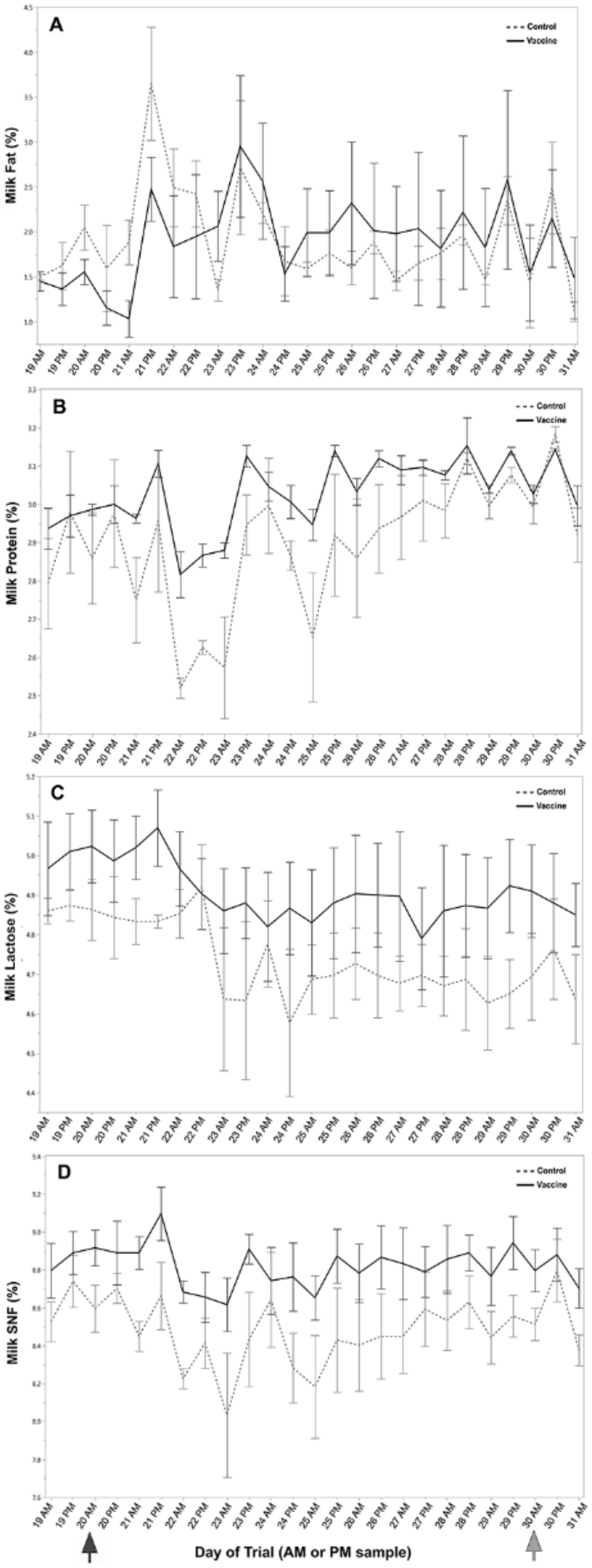

Figure S2. Milk quality assessment during the trial period. **(A)** Percent milk fat, **(B)** milk protein, **(C)** milk lactose, and **(D)** solids-not-fat (SNF). Vaccination occurred on days 1 and 14 (not shown), challenge occurred on day 20 (AM, black arrow) and the end of the challenge period was day 30 (AM, gray arrow).

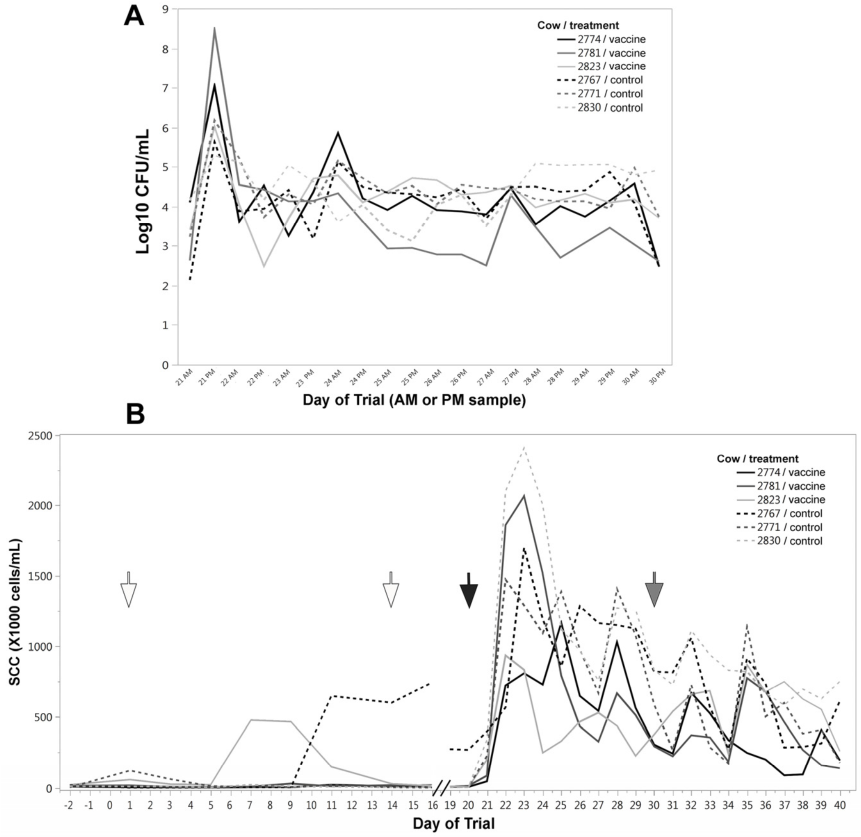

Figure S3. Individual cow bacterial shedding and SCC during trial. **(A)** Quantification of bacterial shedding by cow during the challenge period. Log 10 of CFU/mL of *Staphylococcus aureus* on mannitol salt agar (MSA). **(B)** SCC by cow during entire trial. Dashed lines are control animals and solid lines are vaccinated animals. White arrows indicate vaccination days (1 and 14), dark gray arrow indicates challenge day (20) and light grey arrow indicates the last day of challenge (30).

Scheme 1. Clinical outcomes after challenge on day 20.

Cow	Group	Clinical outcome
2774	Vaccine	No evidence of clinical mastitis
2779	Vaccine	Day 20; severe *Escherichia coli* mastitis in unchallenged quarter (2779 LF) with systemic illness including septicemia. Thereafter, the other three quarters were involved and developed clinical mastitis due to *Staphylococcus aureus* and the animal was euthanized on day 5 post-challenge (day 25)
2781	Vaccine	No evidence of clinical mastitis
2823	Vaccine	No evidence of clinical mastitis, temporary enlargement of supramammary lymph node
2767	Control	Day 24 and day 25; evidence of clinical mastitis in challenged quarters LF and RF
2771	Control	No evidence of clinical mastitis
2830	Control	Persistent clinical mastitis in challenged quarter LR starting on day 23, persistent swelling of supramammary lymph node
